# Residual Neuromuscular Block Remains a Safety Concern for Perioperative Healthcare Professionals: A Comprehensive Review

**DOI:** 10.3390/jcm13030861

**Published:** 2024-02-01

**Authors:** Franziska Elisabeth Blum, Andrew R. Locke, Naveen Nathan, Jeffrey Katz, David Bissing, Mohammed Minhaj, Steven B. Greenberg

**Affiliations:** 1 Department of Internal Medicine, NorthShore University HealthSystem, Evanston, IL 60201, USA; fblum@northshore.org; 2 Department of Anesthesiology, Critical Care, and Pain Medicine, NorthShore University HealthSystem, Evanston, IL 60201, USA

**Keywords:** anesthesia, perioperative care, residual neuromuscular block, quantitative monitoring, neuromuscular blocking agent

## Abstract

Residual neuromuscular block (RNMB) remains a significant safety concern for patients throughout the perioperative period and is still widely under-recognized by perioperative healthcare professionals. Current literature suggests an association between RNMB and an increased risk of postoperative pulmonary complications, a prolonged length of stay in the post anesthesia care unit (PACU), and decreased patient satisfaction. The 2023 American Society of Anesthesiologists Practice Guidelines for Monitoring and Antagonism of Neuromuscular Blockade provide guidance for the use of quantitative neuromuscular monitoring coupled with neuromuscular reversal to recognize and reduce the incidence of RNMB. Using sugammadex for the reversal of neuromuscular block as well as quantitative neuromuscular monitoring to quantify the degree of neuromuscular block may significantly reduce the risk of RNMB among patients undergoing general anesthesia. Studies are forthcoming to investigate how using neuromuscular blocking agent reversal with quantitative monitoring of the neuromuscular block may further improve perioperative patient safety.

## 1. Introduction

**Figure 1:**
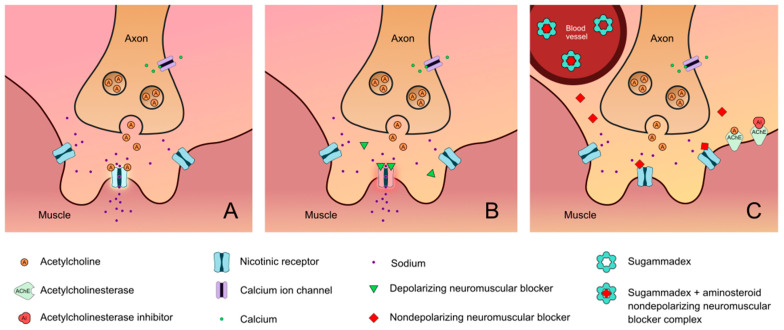
Neuromuscular junction: (**A**) normal; (**B**) activity of depolarizing neuromuscular blocking agent; (**C**) activity of nondepolarizing neuromuscular blocking agent as well as the activity of both neuromuscular blockade reversal agents (i.e., sugammadex and acetylcholinesterase inhibitors) [6,7,8,9,10].

**Figure 2:**
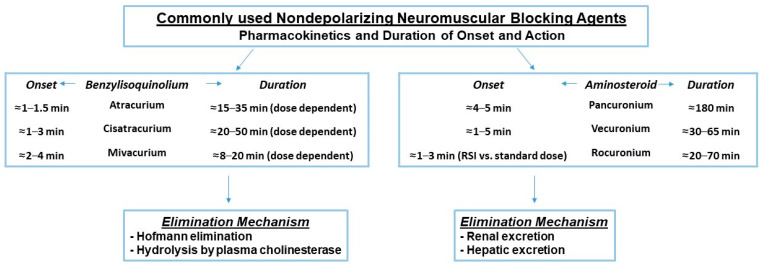
Pharmacokinetics of nondepolarizing agents. Factors that increase the duration of these agents include dosing, age >65 years, hepatic and renal disease, hypothermia, inhaled anesthetics, and medications including but not limited to antibiotics, antidepressants, magnesium, and antiepileptics. The ranges provided for onset and duration times are exclusionary of outliers [11,12,13,14,15,16,17,18,19,20,21,22,23].

Neuromuscular block (NMB) is commonly used in perioperative care for a variety of circumstances. It can optimize surgical conditions [1], facilitate tracheal intubation, and be used in the intensive care unit (ICU) to improve chest wall compliance, reduce abdominal pressure, prevent and treat shivering, and reduce elevation in intracranial pressure from the airway reactivity [2,3]. In the emergency department (ED) and areas outside of the operating room and procedural areas, NMB can be used for rapid sequence induction and intubation (RSII), a technique used by healthcare professionals to minimize pulmonary aspiration [4]. There are two common types of neuromuscular block: depolarizing and nondepolarizing. The only commercially available depolarizing muscle relaxant, suxamethonium chloride or succinylcholine, binds to acetylcholine receptors and causes temporary depolarization. It produces muscle fasciculations followed by flaccid paralysis and is frequently used for urgent or emergent intubation in and outside of the operating room (OR) [4]. Nondepolarizing muscle relaxants are used more frequently in the operating room and provide competitive antagonistic activity of the nicotinic receptor and therefore block the action of acetylcholine (Figure 1) [5]. The nondepolarizing neuromuscular blockers can be further divided into two groups: benzylisoquinolins (or benzylisoquinoliniums) and aminosteroids. Types of drugs within these two classifications have their own unique pharmacokinetic makeup (Figure 2).

**Figure 3:**
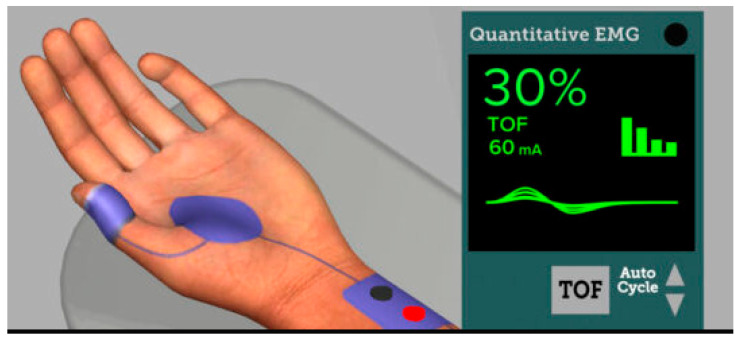
Online simulation course depiction of a quantitative EMG neuromuscular monitor (Adapted with permission from Ref. [37] 2023, APSF).

Residual neuromuscular block (RNMB) is the unwanted presence of signs and symptoms of muscle weakness in the perioperative period and outside of the OR after the administration of neuromuscular blocking agents and is defined by a combination of quantitative and qualitative measures [4,24,25]. To quantify RNMB, a train-of-four ratio (TOFR) recorded on a quantitative neuromuscular monitor was used. The TOFR represents four supramaximal stimuli delivered every 0.5 s (2 Hz) and the muscle response to the fourth stimulus is compared to the first stimulus (Figure 3) [24,26]. A ratio of the fourth to the first response of TOF (train of four) less than 0.90 (90%) defines the RNMB. Although the definition of RNMB has changed over time due to clinical observations of weakness at lower TOFRs, the TOFR < 0.9 is currently the most accurate quantitative definition of residual neuromuscular block.

A quantitative TOFR < 0.9 has been associated with various unwanted symptoms of weakness that include an inability to breathe normally and maintain a patent airway, swallow dysfunction, lack of a strong cough, and even an inability to smile or talk [24,27,28,29,30,31,32]. These undesirable outcomes may be prevented if anesthesia professionals ensure the return of TOFR ≥ 0.9 in the perioperative space [33]. As a result, the widely recommended and accepted TOFR associated with higher rates of full clinical recovery from neuromuscular block is ≥0.9 measured at the adductor pollicis [24,33,34].

While the pharmacodynamic and pharmacokinetic profiles of NMB have improved over the past six decades, along with the monitoring capabilities, nondepolarizing neuromuscular blocking agent (NMBA) use may still result in residual neuromuscular block leading to a variety of deleterious outcomes [24]. Studies have suggested that postoperative pulmonary complications (PPCs) such as pneumonia, respiratory failure, atelectasis, and upper airway obstruction result in increased postoperative mortality and are associated with RNMB [35]. RNMB () has been further associated with prolonged lengths of stay in the post anesthesia care unit (PACU) and decreased patient satisfaction [24,36]. RNMB remains a significant issue in perioperative care and is frequently unrecognized, despite more perioperative provider acknowledgment that this phenomenon exists and the increase in literature on this topic that is now available. This review will discuss the presence of poor recognition of RNMB among anesthesia professionals, the monitoring modalities available, and the special populations that are at highest risk of RNMB. In addition, the review will discuss obstacles to the implementation of quantitative monitoring and the reversal agents that may reduce the incidence of RNMB.

### Poor Recognition of Residual NMB

Despite the rather high reported incidence of RNMB (as high as 65%, in elective abdominal surgeries [38]), many anesthesia professionals are unaware of the clinical significance of RNMB [39,40,41]. Poor recognition of residual neuromuscular block is a driving factor behind this phenomenon [42]. Several international surveys have suggested that anesthesia professionals do not routinely monitor for RNMB as well as underestimating the true incidence of RNMB [27,39]. The main reason for the survey results above may be rooted in the overconfidence of anesthesia professionals in their perceived knowledge of the relevance of neuromuscular monitoring to all aspects of medical practice [27].

## 2. Monitoring

### 2.1. Quantitative NMB Monitoring

The 2023 ASA Practice Guidelines for Monitoring and Antagonism of Neuromuscular Blockade recommend the use of quantitative monitoring to monitor for RNMB as it more reliably allows for the assessment of TOFR ≥ 0.9 (Figure 3) [33] than qualitative measurements. There is a variety of quantitative monitors: acceleromyography (AMG), electromyography (EMG), kinemyography (KMG), and mechanomyography (MMG) [43,44]. EMG technology is favorable in the field given its correlation to MMG results [43,44]. However, AMG has also been used for perioperative monitoring of NMB [32,45].

The target muscle to monitor recovery from NMB is the adductor pollicis muscle innervated by the ulnar nerve. At this site, NMBAs last longer and the muscle recovers more slowly than the diaphragm, laryngeal adductors, and corrugator supercilli in terms of neuromuscular block recovery [46]. Therefore, a TOFR ≥ 0.9 measured at the adductor pollicis is more likely to ensure recovery from NMB. Other areas such as the leg (peroneal) and face (facial nerve) have been used in clinical settings when the arm cannot be used for assessment. However, studies suggest that there are shorter times to recovery with these sites and therefore, these sites may not ensure complete NMB recovery.

### 2.2. Qualitative NMB Monitoring

**Figure 4:**
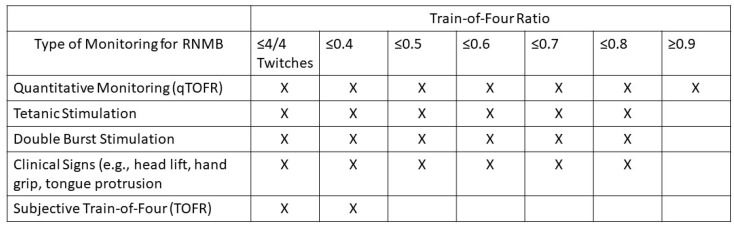
The various types of monitoring and the suggested threshold TOFR equivalent. These TOFR ratio cutoffs are approximate ranges and vary based on different studies performed. The “X” denotes the threshold in which the literature reports reliable detection of the respective TOFR equivalent. Providers should use their own clinical judgement when treating patients [6,34,46,53].

Qualitative monitoring is a subjective form of monitoring of the visual or tactile TOF count (TOFC) or degree of fade in response to stimulation from a peripheral nerve stimulator [43]. There are various methods for conducting qualitative monitoring that include a train-of-four count, tetanic stimulation, double burst stimulation, and sustained tetanus. These forms of qualitative monitoring each have their own unique limitations [47]. Advantages of qualitative monitoring include the ease of observing clinical signs and availability of a peripheral neurostimulator. Qualitative monitoring is subjective and has been associated with signs and symptoms of RNMB, which has been confirmed by a multitude of studies over the years [24,33,47,48,49,50,51]. A recent meta-analysis including 12,664 patients from 53 studies suggested that subjective, qualitative monitoring, and no monitoring were found to be significantly worse than objective, quantitative monitoring with respect to the detection of RNMB (Figure 4) [52].

### 2.3. Clinical Tests

Clinical tests may include, but are not limited to, the 5 s head lift, sustained hand grip, eye-opening, tongue protrusion test, and presence of spontaneous respiration [34,47]. While used widely among anesthesia professionals, these tests lack both sensitivity and specificity for detecting RNMB [43]. Most clinical tests are optimally performed in the awake and cooperative patient, which can be problematic in anesthetized, intubated patients [6]. Furthermore, the presence of these clinical signs does not consistently correlate to upper airway patency and complete recovery of the accessory muscles for breathing [51]. Yet, these tests are still commonly used in practice today [33].

### 2.4. Obstacles to Quantitative Monitoring Implementation

While the use of quantitative monitoring may reduce the incidence of RNMB in the perioperative period, implementation is not without its challenges. The implementation of widespread quantitative monitoring across an institution may be hindered by cost constraints and a significant cultural change, requiring education. Edwards et al. performed an institutional cost analysis of quantitative monitoring which included implementation of 30 monitors for 7500 annual surgical patients. Results suggested five fewer complications related to RNMB annually resulting in no net cost increase [54]. Additionally, there is a cultural barrier to the implementation of institution-wide quantitative monitoring. There is a challenge among anesthesia professionals who are ingrained in their current practice patterns to learn how to operate and interpret information from quantitative monitoring. It has been reported in the literature that physicians have increased difficulty in this exercise of unlearning [55]. A recent multicenter trial investigating the impact of an education intervention program on reducing RNMB found no difference in the incidence of RNMB or use of a reversal agent in the pre- and post-education arms. The study did find a significant increase in the use of quantitative monitoring and a decrease in pulmonary complications [56]. Given the historical lack of effective, commercially available quantitative monitors, clinicians are not often familiar with such devices and the important differences detectable by a quantitative monitor compared to other methods of attempting to monitor residual NMB [34]. Another study found that clinicians lacked an adequate understanding of the adverse clinical outcomes associated with clinical signs and qualitative monitoring [57]. Naguib et al. discuss the importance of convincing clinical providers of the value of monitoring devices as has historically been needed with other anesthesia monitoring devices such as pulse oximetry, capnography, temperature probes, and other standard monitors [34]. Recently, the American Society of Anesthesiologists (ASA) and the Anesthesia Patient Safety Foundation (APSF) released an online simulation course to educate all anesthesia professionals about the perioperative use of quantitative monitoring and appropriate use of reversal agents (https://www.apsf.org/apsf-technology-education-initiative/quantitative-neuromuscular-monitoring/, accessed on 22 November 2023) [37]. This course, coupled with local “champions” who can act as experts and help troubleshoot any issues with the monitors, can enhance education and the implementation of quantitative monitoring with the use of reversal agents and should improve adherence to the recently released guidelines.

## 3. Special Populations

### 3.1. Cardiac and Thoracic Surgery Population

Cardiac and thoracic surgical patients are at particularly high risk of PPCs in the presence of an inadequate reversal of the neuromuscular block (RNMB) [58,59]. Pre-existing pulmonary conditions are prevalent in the cardiothoracic surgical cohort and include chronic obstructive pulmonary disorder (COPD)/emphysema, pulmonary edema, interstitial lung disease, and airway obstruction. The deleterious effects of these underlying conditions are compounded by the sequelae of cardiac and thoracic surgery. Postoperative pulmonary edema, pleural effusions, and impaired respiratory mechanics including reduced functional residual capacity (FRC), decreased lung compliance and impaired cough may all be exacerbated by residual neuromuscular block [60,61].

### 3.2. Morbidly Obese Population

Morbidly obese patients (body mass index (BMI) ≥ 40 kg/m^2^) are also at an increased risk of developing PPCs [62]. The deleterious effects of morbid obesity on respiratory function are numerous. Excessive adipose tissue on the chest wall and intra-abdominal viscera reduce lung compliance and decrease lung volumes (FRC and expiratory reserve volume) [63]. Higher metabolic demand from excess adipose tissue drives a higher minute ventilation, which is achieved by an increased respiratory rate and work of breathing. Reduced FRC increases the risk of postoperative atelectasis and hypoxia [64]. Obesity hypoventilation syndrome in conjunction with the administration of perioperative analgesics increases the incidence of postoperative apnea. All of these processes are exacerbated by inadequate neuromuscular block reversal. Studies evaluating the reversal of neuromuscular block in the morbidly obese suggest that sugammadex may facilitate a faster recovery of the neuromuscular function and may reduce the risk of PPCs when compared to neostigmine [65].

Obese patients may also develop or are at risk of obstructive sleep apnea (OSA), a form of sleep-disordered breathing. A recent meta-analysis including five studies (three observational and two randomized) suggested that OSA patients who receive NMB may be at increased risk of RNMB, hypoxemia, and subsequent respiratory failure [66].

### 3.3. End-Stage Renal Disease (ESRD) Population

Patients with renal dysfunction may also be at increased risk of RNMB [67]. Factors such as reduced elimination of the drugs, accumulation of metabolites, changes in fluid compartment size, alterations in the acid-base balance, decreases in plasma cholinesterase activity, and an increased probability of drug interactions may all place patients with renal disease at increased risk of RNMB. Repeated bolus doses, overdosing, and continuous infusion of aminosteroid NMBs can also increase the risk of RNMB in this patient population [68]. Rocuronium is renally excreted, and the half-life is prolonged in patients with renal disease (Figure 2). It is also important to understand that the sugammadex-rocuronium complex is renally excreted. Currently, the Food and Drug Administration has not approved the use of sugammadex in end-stage renal failure patients with a creatinine clearance less than 30 mL/min [69]. However, the off-label use of sugammadex in this population has been widely reported. Several trials, albeit small, have shown that sugammadex successfully reverses neuromuscular block to a TOFR ≥ 0.9 without evidence of recurarization [70,71,72,73]. A meta-analysis of 655 patients, including 6 prospective (n = 89 ESRD and 90 normal renal function) and three retrospective studies (476 ESRD patients), investigating sugammadex reversal of rocuronium showed a return of the TOFR to 0.9; however, there was a statistically significant slower time to recovery by an average of approximately 75 s in patients with ESRD compared to patients with normal renal function [74]. There were no differences in adverse events in this meta-analysis. Although data suggesting an acceptable safety profile of sugammadex in this population continue to emerge, further investigation may be warranted to fully establish the use of sugammadex in this clinical setting.

The alternative for reversal in this population is the use of neostigmine. However, approximately 50% of its plasma clearance is reliant on renal function. Therefore, it can have a prolonged half-life and reduced clearance in patients with renal failure. Special attention to using adequate doses of antimuscarinics when using neostigmine is important in this population to prevent the prolonged unwanted side effects of neostigmine such as profound bradycardia or heart block [68].

### 3.4. Liver Disease Population

Liver dysfunction may play a role in the altered metabolism of NMB and reversal agents. A study assessing the pharmacokinetics and pharmacodynamics of rocuronium in cirrhotic patients demonstrated a 28% reduction in clearance and a 55% increase in elimination half-life of rocuronium as compared to patients with normal liver function [75]. Abdulatif et al. evaluated neuromuscular block reversal in patients with Childs Class A cirrhosis undergoing liver resection, and demonstrated an 80% reduction in the time to adequate neuromuscular recovery with sugammadex compared to neostigmine [76]. Given these findings, NMBAs should be judiciously dosed and reversed while using quantitative monitoring to mitigate the risk of RNMB.

### 3.5. Neuromuscular Disease Population

Patients with pre-existing neuromuscular disease are at a higher risk of perioperative complications related to neuromuscular block [77] than those without neuromuscular disease. Myasthenia gravis (MG), an autoimmune disease resulting in a decrease in the number of nicotinic receptors at the NMJ, is a disease that is particularly important for the anesthesia professional to understand how to manage perioperatively. These patients are prone to adverse anesthetic outcomes due to RNMB as they suffer from skeletal muscle weakness, which is improved with rest. They are very sensitive to nondepolarizing NMB (NDMB), and therefore short-acting NDMB with the lowest dose used coupled with close neuromuscular monitoring is preferred. Contrary to the sensitivity of NDMB, patients with MG are resistant to succinylcholine, likely due to the loss of nicotinic receptors. A typical dose of succinylcholine used for rapid sequence intubation is 1.5–2 mg/kg. Lastly, patients with MG may be susceptible to a cholinergic crisis resulting from the use of anticholinesterases and the significant increase in acetylcholine at the NMJ. The treatment is typically respiratory support and time. Many anesthesia professionals often allow for complete NMB recovery and may use sugammadex to potentially reduce the risk of this phenomenon [78].

### 3.6. Pediatric Population

The pediatric population experiences different pharmacokinetic and pharmacodynamic profiles of nondepolarizing NMBAs compared to adults [79]. A 2020 study of 6507 pediatric and infant general anesthetics found that high-dose NMBAs were associated with a higher rate of PPCs. This study also suggests that infants, a short surgery duration, and an increased ASA class increase the risk of PPCs from RNMB in pediatric patients [80]. In a 2017 meta-analysis of 580 pediatric patients, Liu et al. found that sugammadex was effective in significantly reducing the time to achieve a TOFR ≥ 0.9. This study also found that in comparison to neostigmine, sugammadex significantly reduced the incidence of bradycardia with no other statistically significant differences in adverse events [79]. The FDA approved sugammadex for expanded use to include the pediatric population (≥2 years old) in 2021 [81].

### 3.7. Geriatric Population

Geriatric patients (patients > 65 years old) may be at greater risk of RNMB compared to adult patients [82]. The aging body experiences a reduction in organ function over time. The effects of steroidal neuromuscular blocking agents, for example, are prolonged in geriatric patients due to the decreased volume of distribution, longer elimination times, changes in circulatory physiology, decreased renal and hepatic blood flow, and anatomic changes in the neuromuscular junction [12]. The result of this is changes in the pharmacodynamic and pharmacokinetic profiles of NMBAs [82] when administered to geriatric patients. [83,84]. Studies suggest that the geriatric population undergoes a significant increase in the clinical duration of NMB as well as more experiencing a TOFR < 0.9 than patients less than 50 years of age [82,85]. Hence, the incidence of RNMB in older patients is suspected to be higher compared to the younger population.

## 4. Prevention of RNMB

Preventing RNMB is paramount to reducing unwanted patient adverse events in the perioperative period. First, a thorough review of the patient’s history, medications, and treatment of reversible conditions could help to prevent RNMB. Second, the patient should also be evaluated for the actual need for NMB. Third, using a shorter-acting NMB while using the minimal amount of NMB necessary to achieve the desired response may reduce RNMB [53]. This approach may lend itself to a higher probability of complete reversal at the end of a surgical case [86]. Lastly, recent literature suggests that sugammadex is superior compared to neostigmine in reducing RNMB [33] and particularly at moderate to deep forms of neuromuscular block. The American Society of Anesthesiologists 2023 Practice Guidelines suggest a systematic approach to implementation that may include quantitative monitoring in all anesthesia locations, individual and departmental education efforts, and performance feedback [33]. Use of the guidelines suggests that complete recovery from RNMB, as monitored through quantitative monitoring, may increase patient satisfaction, decrease PACU length of stay, pulmonary complications, and mortality [33].

### 4.1. Reversal of Neuromuscular Block

#### 4.1.1. Neostigmine

Neostigmine is an acetylcholinesterase inhibitor that has been used for decades to reverse nondepolarizing neuromuscular blocking agents. Its advantages include that it can be used to reverse both classes of NMB, is generally cost-friendly in many countries in the world, and has a very low allergenicity rate [87]. However, it has several disadvantages. First, it requires concomitant use of anticholinergic agents such as glycopyrrolate or atropine to counteract neostigmine’s muscarinic activity that includes bronchospasm, increased gut motility, and bradycardia. Second, according to a recent ASA Practice Guideline it should be used to reverse NMB in the setting of minimal depth of neuromuscular block as defined by a TOFC of equal to 4 without fade (TOFR 0.4–0.9) [33]. It has been shown to be less effective with moderate to deep degrees of block. Third, it takes approximately 10 min to reach maximal effect and, therefore, needs to be administered with this duration kept in mind. Fourth, increasing the neostigmine dose does not always result in enhancing its reversal action due to its ceiling effect. Increasing the dose may also result in a depolarizing block given the increased availability of acetylcholine at the neuromuscular junction. Lastly, it may have a prolonged effect on patients with renal insufficiency [88].

#### 4.1.2. Sugammadex

Sugammadex is a gamma-cyclodextrin that encapsulates aminosteroidal NMB in a 1:1 ratio. It was primarily developed to achieve a more complete recovery from aminosteroidal NMB. The sugammadex structure of eight outer tails that have a negative charge attract the positive charge on the aminosteroid molecule, facilitating an irreversible hold of the NMB in its lipophilic core. The aminosteroid-sugammadex complex is then excreted in the urine [88].

Sugammadex’s drug profile and pharmacokinetics make it an ideal reversal agent for aminosteroid NMBs. First, it does not require an additional agent like an antimuscarinic to counteract its side effects. Second, its fast onset of action can allow rapid reversal even with moderate-deep depths of block within 2 min. With larger doses (4–16 mg/kg), sugammadex can also reverse much deeper forms of NMB than neostigmine. In fact, with the appropriate dosing, sugammadex can reverse any depth of NMB created by rocuronium and vecuronium. Therefore, in situations where profound depths of NMB are used, sugammadex might be the preferred agent when using aminosteroid NMBs [88].

While sugammadex has facilitated a more predictable reversal, especially with moderate to deep depths of NMB, it also has its disadvantages. First, in a variety of countries, the drug may not be available or is too costly for hospitals to purchase. Yet, sugammadex is already released in a generic form in some countries, reducing its cost. Furthermore, cost analyses suggest that when considering the potential for reducing operating room time and adverse effects from RNMB when using sugammadex, especially in the settings of moderate to deeper depths of block, it may either be comparable to neostigmine or reduce overall hospital costs [88]. Second, with any drug shortages, the cost of glycopyrrolate and neostigmine may rival that of sugammadex. Third, it is important to consider all adverse effects of sugammadex, albeit it carries a reasonably low risk. For instance, the risk of anaphylaxis delayed the FDA approval of sugammadex in the US. The incidence has been reported to be anywhere from 0.012–0.0016% [88]. The clinical signs are hypotension, flushing, and bronchospasm. In addition, reports of profound bradycardia leading to cardiac arrest were reported in the literature that seemed not to be connected to anaphylaxis [88].

#### 4.1.3. Perioperative Outcomes Associated with Neostigmine vs. Sugammadex

Several randomized studies have been conducted to date (n − 10) that suggest sugammadex use is associated with a lower incidence of RNMB vs. neostigmine [33]. In addition, several studies suggest that acceptable recovery to a TOF ≥ 0.9 occurs in a shorter period of time with the use of sugammadex vs. neostigmine and in the presence of deep, moderate, and shallow forms of block [33]. However, further studies are required to clearly delineate the benefit with regards to efficacy, safety, and cost when choosing sugammadex vs. neostigmine, particularly in the setting of shallow depths of block. Low strength of evidence suggested a lower incidence of pneumonia with sugammadex vs. neostigmine use [33]. However, when pooling seven non-randomized and six randomized studies, the evidence to date does not suggest a clear difference in overall pulmonary complications (including respiratory failure, hypoxia, infection atelectasis, aspiration pneumonia, bronchospasm or pulmonary edema) with the use of sugammadex vs. neostigmine [33]. Similarly, overall significant differences were not detected among the current studies available that evaluated the presence of tachycardia, bradycardia, hypertension, or arrhythmias when using sugammadex vs. neostigmine (with concomitant use of an antimuscarinic agent). Similarly, there was not an overall significant difference in the incidence of postoperative nausea and vomiting when using sugammadex vs. neostigmine [33].

Given the available evidence, the American Society of Anesthesiologists Practice Guideline was recently released and provides the following overall recommendations for anesthesia professionals with regard to reversal of NMB. A strong recommendation with a moderate level of evidence was provided for the statement that sugammadex should be used over neostigmine to avoid residual neuromuscular block at deep, moderate, and shallow depths of neuromuscular block. With a conditional recommendation with a low level of evidence, the ASA Practice Guideline suggested that neostigmine is a reasonable alternative to sugammadex at minimal depths of NMB. Patients who have achieved a qTOFR ≥ 0.9 do not require any reversal agents. Further studies are forthcoming with regards to comparing patient outcomes with sugammadex and neostigmine at shallow depths of block. In addition, studies should further evaluate the routine avoidance of reversal in patients with qTOFR ≥ 0.9. Therefore, it is suggested that patients who receive neuromuscular blockade during surgery should be reversed unless they achieve the TOF ≥ 0.9. Lastly, further trials investigating the association of RNMB and postoperative pulmonary complications are required and especially in high-risk surgical populations such as those with sleep apnea, morbid obesity, and chronic pulmonary disease [33].

## 5. Conclusions

Residual neuromuscular block continues to affect patient perioperative outcomes. The incidence of RNMB remains unacceptably high. Education and enhancing awareness among all providers using NMB in the perioperative environment may mitigate the occurrence of this phenomenon. The 2023 American Society of Anesthesiologists Practice Guidelines for Monitoring and Antagonism of Neuromuscular Blockade is a major step in providing guidance to all anesthesia professionals. Implementation of quantitative monitoring coupled with the use of reversal agents continues to be a challenge that requires engagement among all perioperative stakeholders in order to achieve success. Further studies are required with regard to identifying the optimal quantitative monitor and whether neostigmine or sugammadex should be used in the setting of shallow block and with the use of aminosteroids. However, one thing is clear and that is that anesthesia professionals can help to reduce perioperative adverse events by eliminating RNMB.

## Abbreviations

AMG	Acceleromyography
APSF	Anesthesia Patient Safety Foundation
ARDS	Acute Respiratory Distress Syndrome
ASA	American Society of Anesthesiologists
COPD	Chronic Obstructive Pulmonary Disease
DBS	Double burst Suppression
ED	Emergency Department
EMG	Electromyography
FDA	Food and Drug Administration
FRC	Functional Residual Capacity
ICU	Intensive Care Unit
KMG	Kinemyography
MG	Myasthenia Gravis
MMG	Mechanomyography
NDMB	Nondepolarizing Neuromuscular Block
NMB	Neuromuscular Block
NMBA	Neuromuscular Blocking Agent
NMJ	Neuromuscular Junction
OR	Operating Room
PACU	Post Anesthesia Care Unit
PPC	Postoperative Pulmonary Complications
PTC	Post Tetanic Count
qTOFR	Quantitative Train-of-four Ratio
RNMB	Residual Neuromuscular Block
TOF	Train-of-four
TOFC	Train-of-four Count
TOFR	Train-of-four Ratio
